# Polygenic risk for coronary artery disease in the Scottish and English population

**DOI:** 10.1186/s12872-021-02398-4

**Published:** 2021-12-07

**Authors:** Chuhua Yang, Fabian Starnecker, Shichao Pang, Zhifen Chen, Ulrich Güldener, Ling Li, Matthias Heinig, Heribert Schunkert

**Affiliations:** 1 Department of Cardiology, Deutsches Herzzentrum München, Technische Universität München, Munich, Germany; 2 Deutsches Zentrum Für Herz- Und Kreislauferkrankungen (DZHK), Partner Site Munich Heart Alliance, Munich, Germany; 3 Medical Graduate Center, Technische Universität München, Munich, Germany; 4 Department of Informatics, Technische Universität München, Munich, Germany; 5 Institute of Computational Biology ICB, Helmholtz Zentrum München (HMGU), Munich, Germany; 6 German Heart Center Munich, Technical University Munich, Lazarettstraße 36, 80636 Munich, Germany

**Keywords:** Coronary artery disease, Prevalence, Genetic risk score, UK Biobank

## Abstract

**Background:**

Epidemiological studies have repeatedly observed a markedly higher risk for coronary artery disease (CAD) in Scotland as compared to England. Up to now, it is unclear whether environmental or genetic factors might explain this phenomenon.

**Methods:**

Using UK Biobank (UKB) data, we assessed CAD risk, based on the Framingham risk score (FRS) and common genetic variants, to explore the respective contribution to CAD prevalence in Scotland (n = 31,963) and England (n = 317,889). We calculated FRS based on sex, age, body mass index (BMI), total cholesterol (TC), high density lipoprotein cholesterol (HDL-C), systolic blood pressure (SBP), antihypertensive medication, smoking status, and diabetes. We determined the allele frequency of published genome-wide significant risk CAD alleles and a weighted genetic risk score (wGRS) for quantifying genetic CAD risk.

**Results:**

Prevalence of CAD was 16% higher in Scotland as compared to England (8.98% vs. 7.68%, *P* < 0.001). However, the FRS only predicted a marginally higher CAD risk (less than 1%) in Scotland (12.5 ± 10.5 vs.12.6 ± 10.6, *P* = 0.03). Likewise, the overall number of genome-wide significant variants affecting CAD risk (157.6 ± 7.7 and 157.5 ± 7.7; *P* = 0.12) and a wGRS for CAD (2.49 ± 0.25 in both populations, *P* = 0.14) were remarkably similar in the English and Scottish population. Interestingly, we observed substantial differences in the allele frequencies of individual risk variants. Of the previously described 163 genome-wide significant variants studied here, 35 variants had higher frequencies in Scotland, whereas 37 had higher frequencies in England (*P* < 0.001 each).

**Conclusions:**

Neither the traditional risk factors included in the FRS nor a genetic risk score (GRS) based on established common risk alleles explained the higher CAD prevalence in Scotland. However, we observed marked differences in the distribution of individual risk alleles, which emphasizes that even geographically and ethnically closely related populations may display relevant differences in the genetic architecture of a common disease.

**Supplementary Information:**

The online version contains supplementary material available at 10.1186/s12872-021-02398-4.

## Introduction

CAD is one of the most prevalent complex diseases [1]. Its pathogenesis is influenced by an interplay of genetics, diet, lifestyle, environmental and socioeconomic factors [2]. Regional differences in CAD prevalence have been observed globally, continentally and even among adjacent countries. For example, as compared to the Netherlands and the United Kingdom (UK), Spain had a constantly lower CAD rate throughout the past 20 years [3]. The same phenomenon can be observed among populations within the UK. In the last 15 years Scotland had constantly higher CAD prevalence compared to England, Wales, and Northern Ireland—the underlying reasons being largely unclear [4].

In principle, both environmental factors or genetics may contribute to the local disparities in CAD [5, 6]. Compared to the European population, Burokienė et al. found that high BMI and poor plasma lipid profiles are primarily responsible for higher cardiovascular disease (CVD) mortality in Lithuania whereas no difference was found for a genetic risk score based on 60 CVD-associated Single-nucleotide polymorphisms (SNPs) [7]. Indeed, exogenous risk factors affected by culture, lifestyle, or socioeconomics can undergo rapid changes on the individual, familial, and population level leading to marked temporal changes in CAD prevalence [6, 8].

Evolutionary genetics determine the allele frequency in a population, which is modulated by natural selection and stochastic forces such as genet drift [9]. These and other factors contribute to variation among individuals in the same population and across populations [8–10]. While mutations causing monogenic disorders are under evolutionary pressure, this applies, to a lesser extent to, common risk alleles with small effect sizes [10]. Indeed, genome-wide association studies (GWAS) revealed that most common cardiometabolic conditions like hypertension, diabetes mellitus, or hyperlipidemia are affected by hundreds of risk alleles, most of which are common [11]. The high number of susceptibility variants and their high allele frequencies jointly contribute to the genetic architecture of disease [9, 12].

Lately, genetic risk scoring has been found to be useful in CAD risk prediction as well as therapeutic and lifestyle guidance. Using a GRS based on 27 SNPs, Mega et al. observed that individuals at high genetic risk have greater benefit from statin therapy [13]. Moreover, Khera et al. showed that a healthy lifestyle drastically reduces risk of incident CAD events among individuals at high genetic risk [14]. Besides for individual disease risk prediction, GRS are also used to assess and compare the risk allele burden between populations with different disease prevalence. Keaton et al. found ethnic-specific differences in the genetic architecture in the context of type 2 diabetes (T2D) between African- and European-Americans [15], whereas Werissa et al. found no such difference between the Roma and the Hungarian general population [16]. Pima Indians in Arizona have the highest prevalence and incidence of non-insulin-dependent diabetes of any geographically defined population [17], but Hanson et al. found that this is not attributable to allele frequency differences at 63 diabetes loci [18].

In this study, we explored whether the higher CAD prevalence in the Scottish population could be explained by traditional risk factors and / or common genetic variants. We used a traditional scoring model, the FRS, and a GRS model based on 163 established common risk alleles.

## Materials and methods

### UK Biobank population

UK Biobank (https://www.ukbiobank.ac.uk/) is a powerful prospective cohort study resource of ~ 500,000 volunteer participants originating from Great Britain. Genome-wide genotyping and various phenotypic data are available on nearly every participant [19]. At recruitment, participants answered a series of questions on lifestyle, health-related information and socio-demographics, and received a range of physical measures, which can be obtained by researchers. After quality control including filtering for sex discordance, missingness, heterozygosity, kinship coefficient and ethnic background, our study contained 442,860 individuals with eligible genotype data. Based on their country of birth, they were grouped into England, Northern Ireland, Wales, and Scotland (Additional file 1: Fig. 1, Table 1).

Baseline characteristics were recorded in the assessment centers of UK Biobank, such as age, gender, BMI, SBP, HDL-C, TC, and smoking status. Lifestyle and environment factors, as well as family history and ethnic background were self-reported. Medications and treatments were collected by questioning. To include more samples, we combined the UK Biobank data fields 20,003, 6177 and 6153 to extract antihypertensive drugs for each individual and used the same strategy to identify CAD and diabetes. All variables used in the FRS are available in Additional file 1: Table 2. All variables used in the QRISK 3 score are available in Additional file 1: Table 3. The diagnosis codes used to identify cases and the medication codes used to identify antihypertensives can be found in Additional file 1: Tables 4 and 5.

In order to keep measured factors consistent with age (reported at the first visit) for Framingham risk score calculation, we used only first recorded value (instance 0 data) from UK Biobank, at which participants were recruited from 2006 to 2010. The CAD prevalence and sample size can be found in Additional file 1: Table 1, and 371,077 individuals had both complete phenotype data and eligible genotype data (Additional file 1: Fig. 1, Table 6).

The study was approved by the Research Tissue Bank (RTB) and the National Research Ethics Service and UK Biobank’s governing Research Ethics Committee (REC), and was conducted in accordance with the principles of the UK Biobank Ethics Advisory Committee (EAC).

### Source of CAD-associated SNPs

Based on a review by Erdmann et al., we extracted lead SNPs of 163 CAD risk loci with genome-wide significance as derived from the decade of GWAS [20]. All 163 CAD-associated SNPs had odds ratios > 1.03 (Additional file 1: Table 7) and were based on different individual studies using genotype data on 4,000,000 variants of more than 100,000 individuals. Besides, Khera et al. generated a CAD polygenetic risk score (PRS) including 6.6 million common genetic variants using a Bayesian approach called the LDpred algorithm, which uses an linkage disequilibrium (LD) reference panel to infer correlation patterns between SNPs for PRS calculations [21]. This genome-wide CAD PRS has more robust *P* values and higher effect estimates on CAD prevalent and incidence [22].

### Calculation of the Framingham risk score and QRISK3 score

From the perspective of mathematical modeling, the Framingham risk prediction algorithm was developed and validated in large community-based samples, and the score is calculated by summing up all risk factors weighted by their estimated regression coefficients from Cox proportional hazards models for women and men separately [23]. The variables required to estimate the 10-year CVD risk include age, gender, SBP, HDL-C, TC, antihypertensive use, smoking status, and diabetes status, which are all available in UKB datasets. There are two models to calculate the FRS. The primary one uses lipids (HDL-C and TC), and the simpler one uses the BMI instead. The codes used to calculate the FRS_lipids and FRS_BMI can be found in Additional file 1: Table 10.

Hippisley-Cox et al. have developed the QRISK3 prediction algorithm that underlies 10-year cardiovascular disease risk in men and women [24]. The primary care systems presently recommend to prescribe statins to individuals with a QRISK3 risk score more than or equal to 10%, according to the current guidelines in Wales and England [25, 26]. Therefore, we consider the QRISK3 score as an alternative to estimate the CAD risk. We included 199,778 individuals without missing data in any of the 22 QRISK3 variables, born in England and Scotland with genotype data (Additional file 1: Tables 3, 8). We used R Package QRISK3 (version 0.3.0) [24, 27] to calculate the 10-year CVD risk score for each individual. All 22 variables used in QRISK3 algorithm were available in UK Biobank Assessment Centre. Lifestyle, environment and family history were self-report, such as smoking and ethnic background. Medication and treatment were collected by verbal interview, such as corticosteroid use and antihypertensives treatment.

### Computation of uGRS and wGRS

After the exclusion of individuals with any missing phenotype data and genotype data of poor quality, we calculated the unweighted (uGRS, the raw counts or the number of risk alleles) and weighted (wGRS) genetic risk scores to assess whether the genetic risk at population level is different between England and Scotland.

1 $$GRS=\sum_{k=1}^{K}{b}_{k}{X}_{k}$$

In the SNP-based additive polygenic genetic model [28], Eq. (1), let X_1_, …, X_k_ denotes the number of risk alleles of SNP k in one individual, and let b_1_, …, b_k_ denote the weight of SNP k. X_k_ = 0 indicates no risk allele, while heterozygotes for the risk allele were coded as genotype X_k_ = 1 and homozygotes for the risk allele as genotype X_k_ = 2. Missing genotypes were imputed by their expected value, which is twice of the risk allele frequency in the population. Therefore, the effects of risk alleles at all loci are regarded as the same if all b_k_ equal 1. In this case a person's summary genetic risk score is the sum of all risk alleles at all loci, which is denoted as uGRS. Giving distinct weights to risk alleles of each SNP, alleles with larger effect size contribute more to the GRS, and wGRS is the sum of the number of risk alleles multiplied the corresponding log odds ratio of each risk allele. Additional file 1: Table 7 indicates the SNPs and risk alleles identified in independent GWAS studies [20], which are used for the uGRS as well as the log odds ratios for the wGRS.

### Statistical methods

We used Pearson's chi-squared test to determine the significance of the difference in CAD prevalence between the two populations. The difference between two populations in FRS and QRISK3 were tested by two tail Mann–Whitney test as both are skewed and not normal distributions. The difference of means of the number of risk alleles and wGRS were assessed by two tail t-test as both are approximately normally distributed. Their distribution comparison was assessed by the Kolmogorov–Smirnov test. We used R version 4.0.3 with packages such as data.table [29], epiR, ggplot2, Table 1, and tidyverse for data analysis and plotting. PLINK2 was used to calculate uGRS and wGRS. PRSice-2 (Polygenic Risk Score Software for Biobank-Scale Data) was used to calculate a wGRS derived from 6.6 million variants [22]. The difference of risk alleles frequencies (RAF) between two populations are tested by Pearson's chi-squared test, and we adjust these *p*-values for multiple comparisons by Bonferroni correction. We used a significance level of *P* < 0.05 for the means and distribution tests.

**Table 1:** Basic Characteristics of participants born in England and Scotland in UK Biobank

	England (N = 317,889)	Scotland (N = 31,963)	*P*-value
Gender***			0.0002
F	169,679 (53.4%)	17,411 (54.5%)	
M	148,210 (46.6%)	14,552 (45.5%)	
*Age (years)*			0.0519
Mean (SD)	56.7 (± 8.1)	56.6 (± 8.0)	
BMI***			0.0002
Mean (SD)	27.4 (± 4.7)	27.49 (± 4.7)	
*HDL cholesterol (mg/dL)*			0.1296
Mean (SD)	26.2 (± 6.9)	26.1 (± 6.9)	
Total cholesterol (mg/dL)*			0.0381
Mean (SD)	102.8 (± 20.6)	103.1 (± 20.7)	
Systolic blood pressure (mmHg)***			< 0.001
Mean (SD)	138.0 (± 18.5)	139.0 (± 18.9)	
Antihypertensive medication**			0.0012
Yes	72,816 (22.9%)	7,577 (23.7%)	
No	245,073 (77.1%)	24,386 (76.3%)	
Smoking***			< 0.001
Yes	32,484 (10.2%)	4,078 (12.8%)	
No	285,405 (89.8%)	27,885 (87.2%)	
Diabetes***			< 0.001
Yes	24,646 (7.8%)	1,918 (6.0%)	
No	293,243 (92.3%)	30,045 (94.0%)	

*SD: standard deviation; BMI: body mass index; HDL: high-density lipoprotein*

^*^p < 0.05; **p < 0.01; ***p < *0.001*

## Results

### Baseline characteristics of study participants

**Fig. 1:**
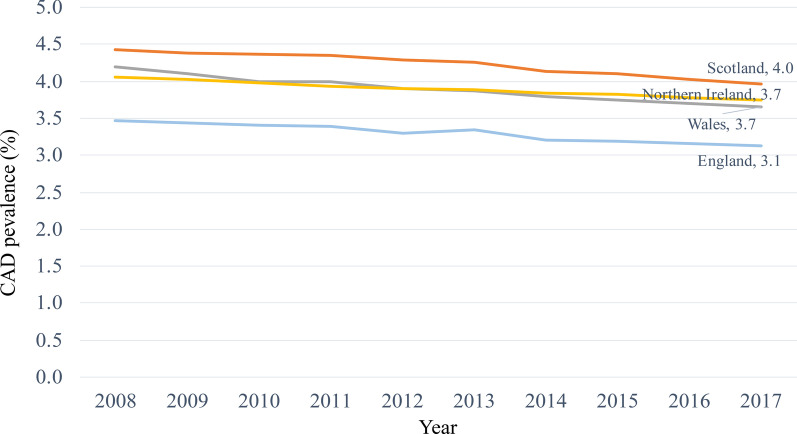
Trends in the CAD prevalence from QOF data, England, Wales, Scotland, and Northern Ireland 2008 to 2017. *Source*: England—Health and Social Care Information Centre. QOF achievement data; Scotland—ISD Scotland. QOF achievement data to 2015/16. Prevalence data for 2016/17 and 2017/18 obtained via personal communication; Wales— StatsWales. QOF achievement data; Northern Ireland—Department of Health, Social Services and Public Safety. QOF exception reporting data 2017/18; QOF, Quality and Outcomes Framework [4]

After exclusion of participants with missing covariates required for calculation of GRS or FRS, we obtained a set of 371,077 samples fulfilling our study requirements. The prevalence of CAD within UKB was highest in Scotland, followed by Wales, Northern Ireland, and England, which matches respective trends in published data from 2008 to 2010 [4] (Fig. 1**,** Additional file 1: Table 6). After data filtering, a significant difference in CAD prevalence was observed between England (n = 317,889; 7.68%) and Scotland (n = 31,963; 8.98%, *P* < 0.001), as well as between England and Wales (n = 18,724; 8.30%, *P* = 0.002), while there was no significant difference between England and Northern Ireland (n = 2,501; 8.36%, *P* = 0.20). Considering the well-established difference in CAD prevalence between Scotland and England, we focused our comparison on these two populations.

The English and Scottish participants had a similar mean age (56.7 ± 8.1 in England and 56.6 ± 8.0 in Scotland) (Table 1). Among traditional CAD risk factors, the Scottish had moderate, but significantly higher levels of BMI and SBP (*P* < 0.001). There were also more smokers, but less diabetics among the Scottish compared to the English population (*P* < 0.001) (Table 1**)**.

### Comparison of traditional risk factors by the Framingham risk score and QRISK3 score

**Fig. 2:**
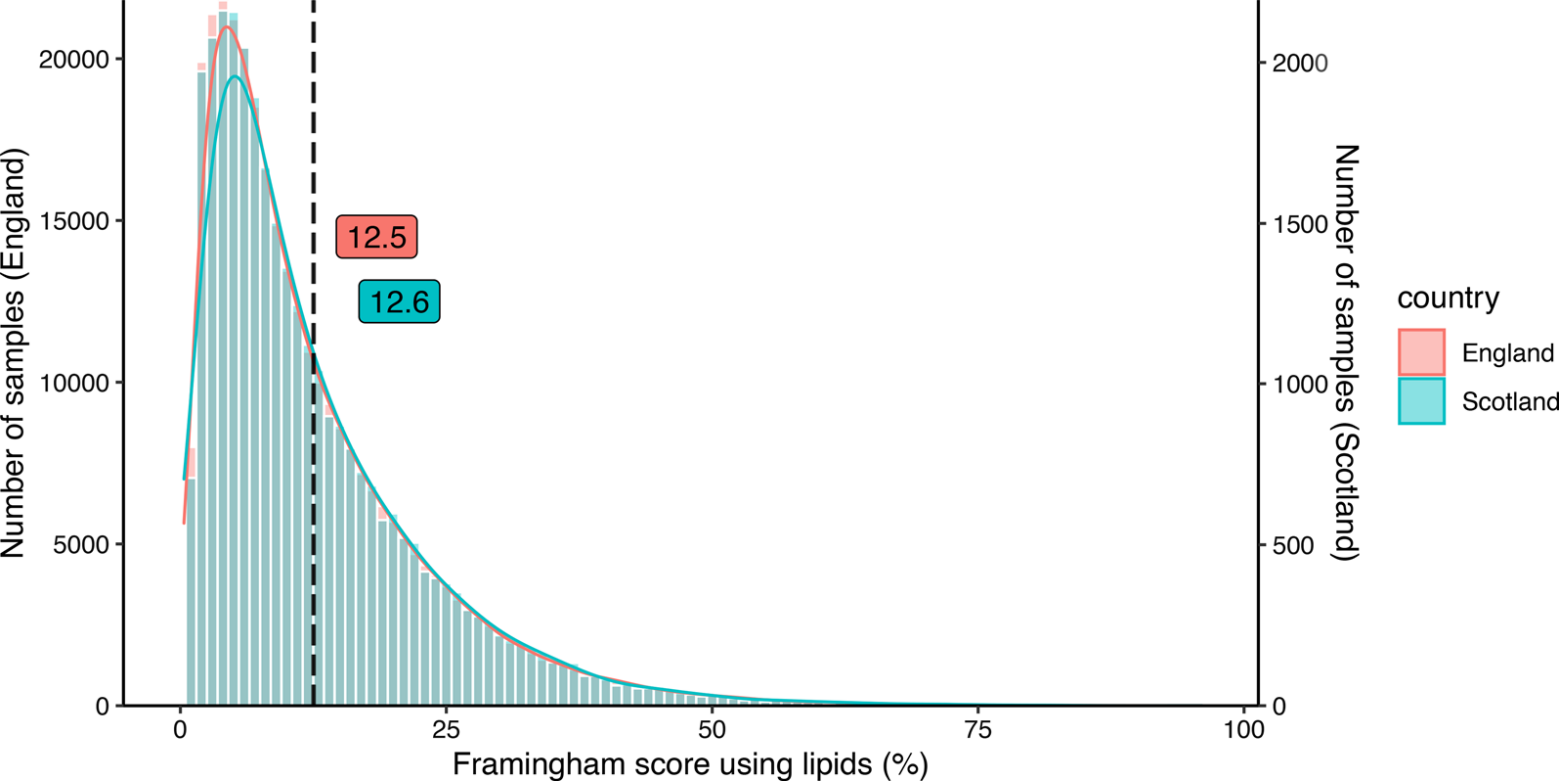
Histograms showing the distribution of the Framingham score using lipids for the comparison population (born in Scotland) and the reference population (born in England)

FRS_lipidsranged from 0.5 to 94.7 (mean, 12.6 ± 10.6) for the Scottish, and from 0.3 to 96.1 (mean, 12.5 ± 10.5) for the English population (*P* = 0.009; Table 2, Fig. 2). Thus, the FRS explains a difference of CAD prevalence of less than 1% whereas the observed prevalence differed by 16.9% between the two countries. Computing the FRS_BMI instead of lipids yielded similar results (Additional file 1: Fig. 2, Table 9). Likewise, estimation of CAD risk based on QRISK3 revealed only small but statistically significant differences between the two countries (Additional file 1: Fig. 3, Table 9**)**.

**Table 2:** Statistics for the Framingham score and genetic risk score in populations

	England (N = 317,889)	Scotland (N = 31,963)	*P*-value
Framingham score using lipids**			0.009
Mean (SD)	12.5 (10.5)	12.6 (10.6)	
Median [Min, Max]	9.2 [0.3, 96.1]	9.4 [0.5, 94.7]	
*Coronary artery disease uGRS*			0.1173
Mean (SD)	157.6 (7.7)	157.5 (7.7)	
Median [Min, Max]	157.6 [122.1, 196.0]	158.0 [122.6, 186.7]	
*Coronary artery disease wGRS*			0.1419
Mean (SD)	10.6 (0.5)	10.6 (0.5)	
Median [Min, Max]	10.6 [8.0, 13.0]	10.6 [8.0, 12.7]	

SD: standard deviation

**p < 0.01

### Comparison of the genetic burden by the polygenic risk score

**Fig. 3:**
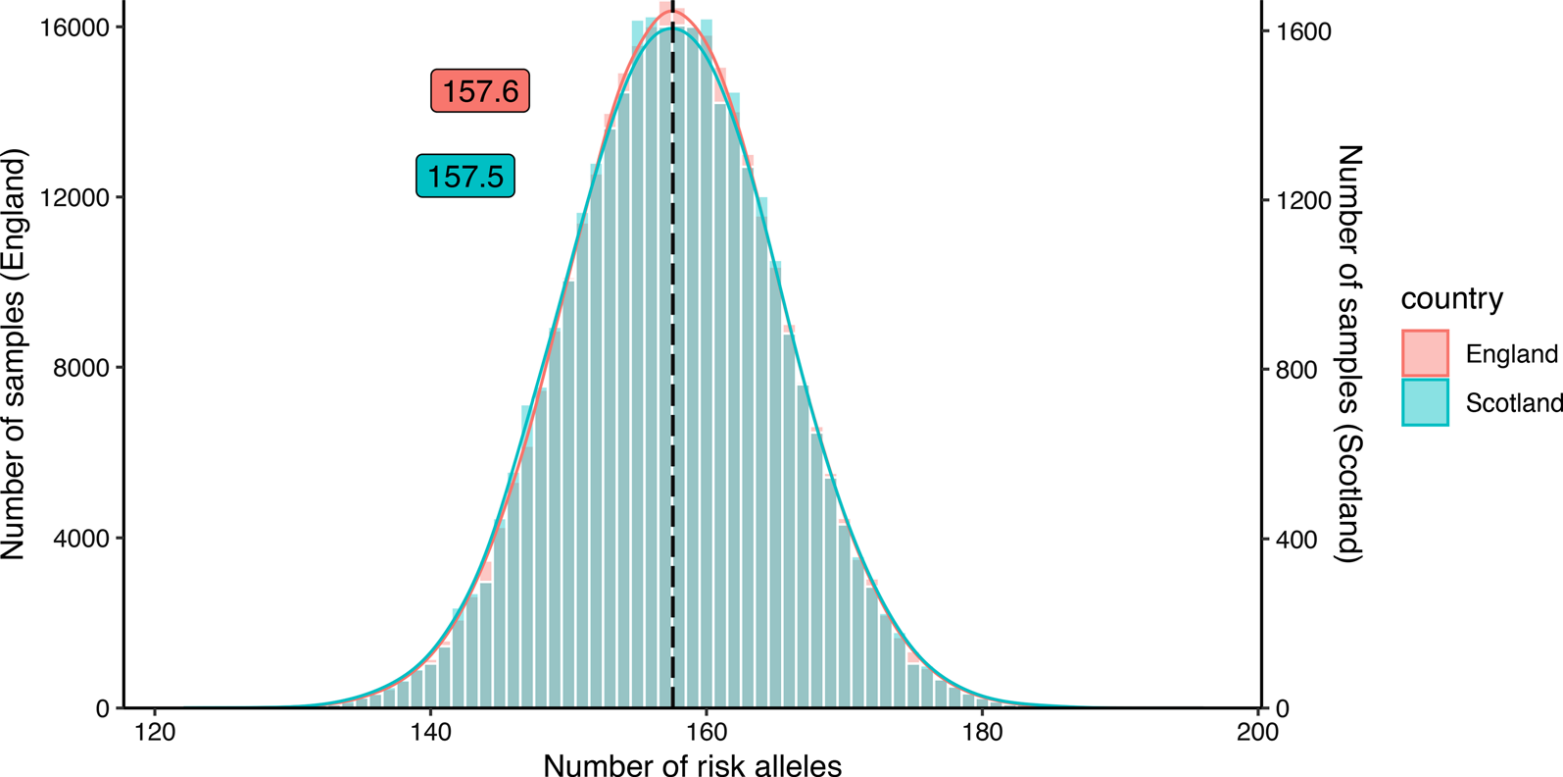
Histograms showing the distribution of the number of risk alleles based on 163 CAD associated SNPs for the comparison population (born in Scotland) and the reference population (born in England)

To investigate whether common genetic variants might predict the higher CAD prevalence in Scotland, we compared the population-based CAD GRS of Scotland and England based on 163 GWAS SNPs significantly associated with CAD (GWAS P < 5E−8, OR > 1.03) [20]. On average, Scottish participants had 157.5 ± 7.7 risk alleles while English individuals had 157.6 ± 7.7 (Fig. 3, Table 2). Both, mean and distribution of uGRS based on 163 SNPs showed no significant difference between the two countries (Table 2). The same result was observed for wGRS based on CAD-associated SNPs. Namely, both countries had a mean wGRS of 10.6 and no difference in wGRS distribution of the two populations was observable (Table 2, Additional file 1: Fig. 4). Finally, CAD risk based on a GRS derived from 6.6 million variants [21]^22^ revealed no differences between the two countries (Additional file 1: Fig. 5, Table 9).

### Comparison of risk allele frequencies

**Fig. 4:**
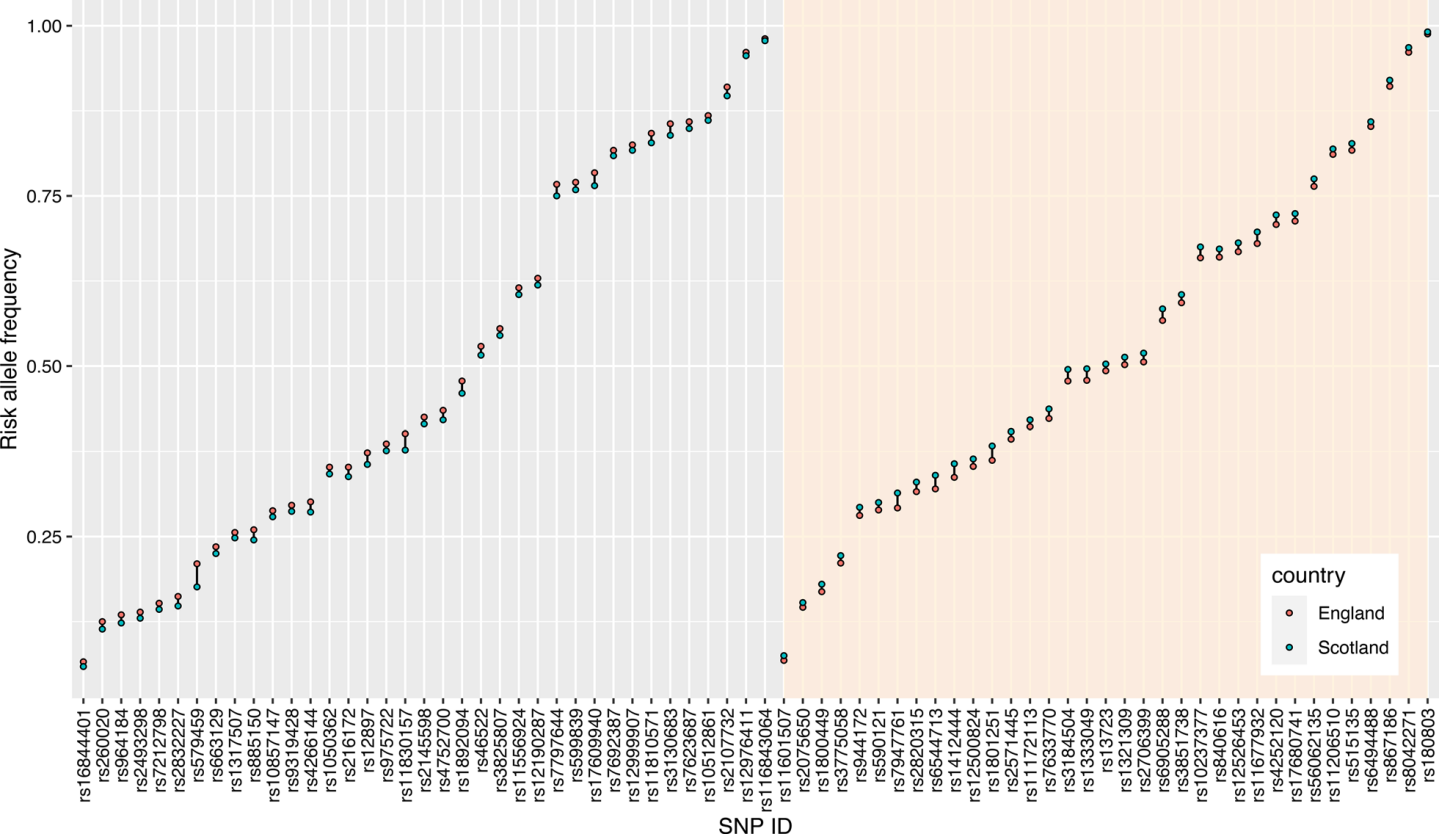
Dumbbell plot showing the risk allele frequency per SNP in England and Scotland. The left gray block shows the 37 SNPs with higher allele frequencies in England (*P* < 0.001). The right antique white block shows the 35 SNPs with higher allele frequencies in Scotland (*P* < 0.001). Non-significant SNPs are shown in the Additional file 1: Fig. 6

We next calculated the risk allele frequency (RAF) at 163 loci with established genome-wide significant association with CAD in England and Scotland (Fig. 4, Additional file 1: Fig. 6, Table 7). There were 35 variants with higher RAF in Scotland whereas 37 had higher RAF in England (Fig. 4, P_adjust_ < 0.001 each). The absolute difference in RAF ranged from 0.3% (rs116843064, England = 98.1%, Scotland = 97.8%) to 3.3% (rs579459, England = 21.0%, Scotland = 17.6%). As mentioned above, these differences neutralized each other since the GRS displayed no significant differences between the two countries.

## Discussion

The prevalence of CAD is higher in Scotland than in England for largely unexplained reasons [4, 30]. This observation was also evident in the UK Biobank participants studied here. The traditional risk factors included in the FRS hardly explained the difference in CAD prevalence between the two countries. Out of 163 genome-wide significant risk alleles studied, 35 had higher RAF in Scotland whereas 37 had higher RAF in England. However, overall, these differences appeared to neutralize each other since there was no significant difference in the means and distributions of both weighted and unweighted GRS based on 163 CAD SNPs.

According to the ancestral-complex disease susceptibility model, genetic variations existed before the human spreading out of Africa and evolved with an extremely slow speed [31, 32]. However, nowadays environment and lifestyle are remarkably different from that of our ancestors. A mismatch between the ancestral variants and current environment might contribute to the development of some of non-communicable, complex diseases [2, 33].

It is unclear as to whether differences in ancestral variants contributing to CAD risk explain regional differences in CAD prevalence. With respect to England and Scotland, we observed that about 40% of genome-wide significant variants displayed significant differences in allele frequencies. It is remarkable to find that many significant differences in allele frequencies of disease relevant genes in such closely related populations. However, the balanced effect—35 variants had higher RAF in Scotland and 37 had higher RAF in England—suggests that this is not driven by any selection pressure on these risk alleles, which is in line with findings of Keyue and Iftikhar, who did not observe significant differences in the distribution of Fst values at 158 CVD-associated SNPs compared to background SNPs [34]. In fact, the net effects of these differences at multiple loci seem to neutralize each other, since we observed no differences in the CAD risk based on polygenic risk scores.

Thus, genetic susceptibility to CAD—based to common risk alleles—appears to be rather similar in England and Scotland. The same applies to traditional risk factors for CAD, since the present as well as previous studies failed to demonstrate profound differences between these two countries [35, 36]. In 1989, Carstairs and Morris reported that Scotland suffers from more severe deprivation than England and Wales [37], In 2011, the same pattern of deprivation was still observed between the countries of Scotland and England [38]. In 2013, Newton et al. reported that significant health inequalities remain between the poorest and most deprived areas [39]. Thus, social deprivation might be one of the explanations for Scotland´s higher CAD rates. In order to lower CAD rates in Scotland, it seems to be reasonable to intensify preventive measures to be delivered at the most deprived.

A limitation of our study may be the fact that the lead SNPs we used to represent risk at a given genome-wide significant locus might not be the causal ones. However, these variants were associated with the strongest risk such that the causal variants are likely to be in very high LD. Moreover, the estimation of risk based on polygenic risk scores is unlikely to be affected by lack of knowledge on the causal variant. Another limitation of our study could be that we did not explore rare variants, gene–gene interactions, gene-environment, and exposure to epigenetic factors. All of these can modulate genetic risk [2, 40, 41] but are challenging to investigate in a study like ours. As for the traditional factors analysis, we only included the major risk factors for CAD (sex, age, BMI, HDL-C, TC, SBP, antihypertensive medication, smoking status and diabetes), while other important factors such as physical activity, family history and socioeconomic status are not included in the Framingham risk model [42]. Finally, the UKB population has been considered to represent a relatively low risk. As such, the data may not be representative for the entire population spectrum [43]. Nevertheless, the repeatedly observed differences in CAD prevalence between Scotland and England were apparent in UKB as well.

## Conclusions

Using representative data from UK Biobank, our study assessed traditional and genetic risk models for discrimination of CAD prevalence in Scotland and England. Our study found that the traditional risk factors included in FRS may explain little of the difference in CAD prevalence between Scotland and England. Likewise, both unweighted and weighted GRS based on 163 SNPs or 6.6 million SNPs suggested a similar genetic susceptibility to CAD in the Scottish and English populations. Yet, there have to be reasons why the Scottish population constantly has higher CAD rates than the English. If genetics can´t elucidate this observation, environmental or lifestyle factors that have received less attention thus far might provide an answer [44–47].

## Supplementary Information

**Additional file 1.** Flowcharts, data preparation, results and code, which are all designed, tidied and calculated by ourselves.

## Data Availability

The datasets analyzed during the current study are available in UK Biobank: https://www.ukbiobank.ac.uk/. The codes used to calculate the FRS_lipids and FRS_BMI can be found in Additional file 1. R package for analysis: https://www.bioconductor.org/

## Abbreviations

CAD: Coronary artery disease
UK: United Kingdom
FRS: Framingham risk score
GRS: Genetic risk score
uGRS: Unweighted genetic risk score
wGRS: Weighted genetic risk score
CVD: Cardiovascular disease
SNP: Single nucleotide polymorphism
GWAS: Genome-wide association studies
BMI: Body mass index
SBP: Systolic blood pressure
HDL-C: Low Density Lipoprotein-Cholesterin
LD: Linkage disequilibrium
PRS: Polygenetic risk score
TC: Total cholesterol
FRS_lipids: Framingham risk score using lipids
FRS_BMI: Framingham risk score using BMI
UKB: UK Biobank
RAF: Risk allele frequency
