# Supplementary material for: Polygenic risk for coronary artery disease in the Scottish and English population
Source: BMC Cardiovasc Disord. 2021 Dec 7;21:586. doi: 10.1186/s12872-021-02398-4 (PMC8650538; doi:10.1186/s12872-021-02398-4)
Supplement: Supplementary file 1 — Additional file 1. Flowcharts, data preparation, results and code, which are all designed, tidied and calculated by ourselves. [file 12872_2021_2398_MOESM1_ESM.pdf]

Table of Contents

Supplementary Figures *2*

Supplementary Fig. 1 Filtering flow chart including qualified individuals for analysis 2

Supplementary Fig. 2 Histograms showing the distribution of the Framingham score using BMI for comparison population (born in Scotland) and reference population (born in England). 3

Supplementary Fig. 3 Histograms showing the distribution of the QRISK3 score for comparison population (born in Scotland) and reference population (born in England). 3

Supplementary Fig. 4 The density plot showing the distribution curves of weighted genetic risk score based on 163 CAD-associated SNPs for comparison population (born in Scotland) and reference population (born in England). 4

Supplementary Fig. 5 The density plot showing the distribution curves of weighted genetic risk score based on 6.6 million CAD-associated SNPs for comparison population (born in Scotland) and reference population (born in England). 4

Supplementary Fig. 6 Dumbbell plot showing the risk allele frequency per SNP in England and Scotland. There was no significant difference between England and Scotland in risk allele frequency among 91 variants. 5

Supplementary Tables *6*

Supplementary Table 1 Sample size with eligible genotype data and CAD prevalence in 4 countries of UK 6

Supplementary Table 2 Variables used when calculating the Framingham risk scores for Englander and Scottish individuals in UK Biobank at baseline 6

Supplementary Table 3 Variables used when calculating QRISK3 scores for Englander and Scottish individuals in UK Biobank at baseline 7

Supplementary Table 4 Code list for antihypertensives in Data-Field 20003 8

Supplementary Table 5 Codes list for identification of CAD 9

Supplementary Table 6 Sample size with both eligible genotype data and variables for Framingham score and CAD prevalence in 4 countries of UK 12

Supplementary Table 7 Comparison of the risk allele frequencies across 163 CAD-associated SNPs between England and Scotland 12

Supplementary Table 8 Characteristics of individuals aged 39-74 in two sub-cohorts from UK Biobank for calculating QRISK3 score 17

Supplementary Table 9 Statistics for the Framingham score, QRISK3 score and genetic risk score in populations 18

Supplementary Table 10 Script written for calculating the Framingham risk score using lipids and BMI 19

Supplementary Figures

Supplementary Fig. 1 Filtering flow chart including qualified individuals for analysis

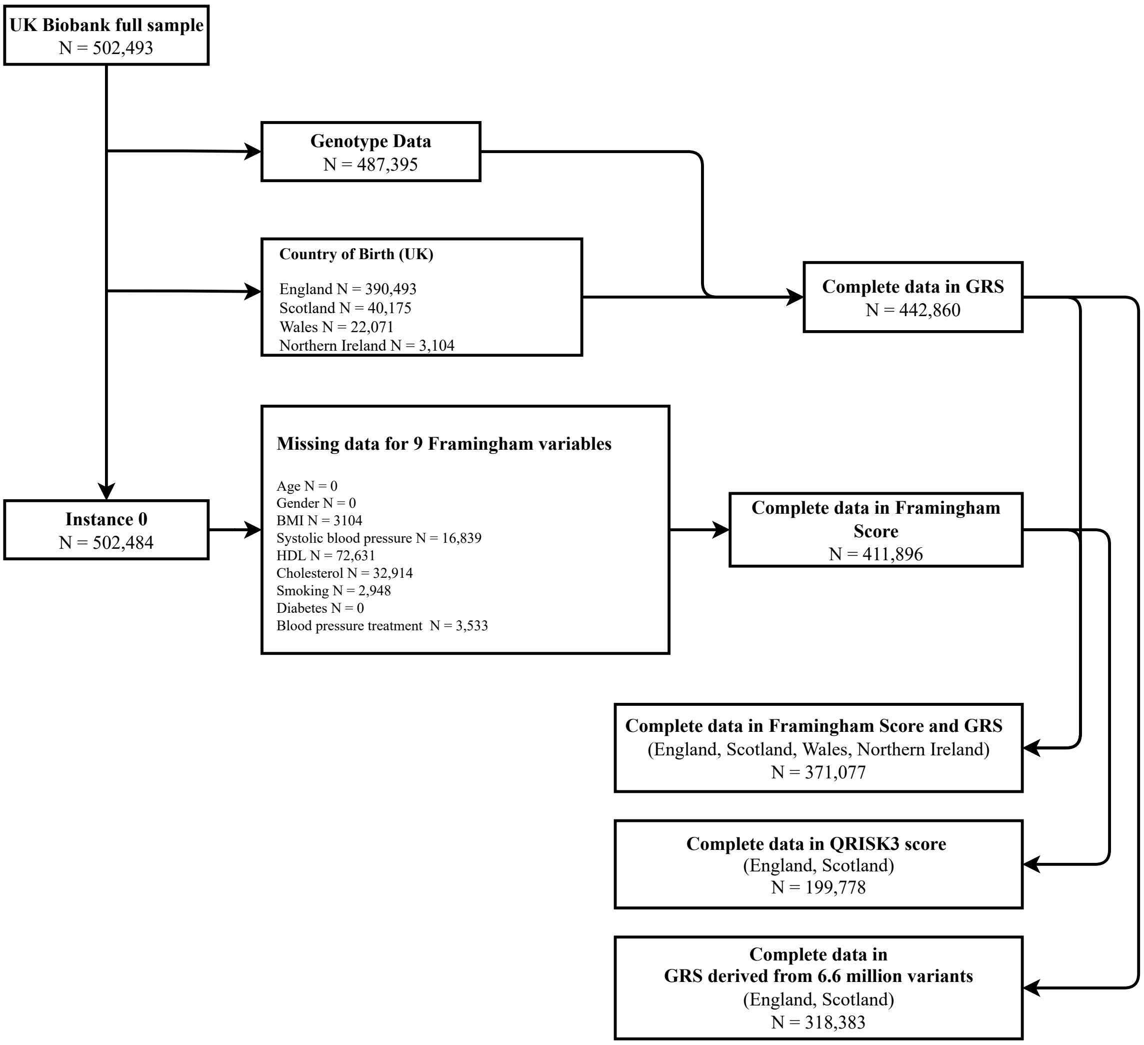

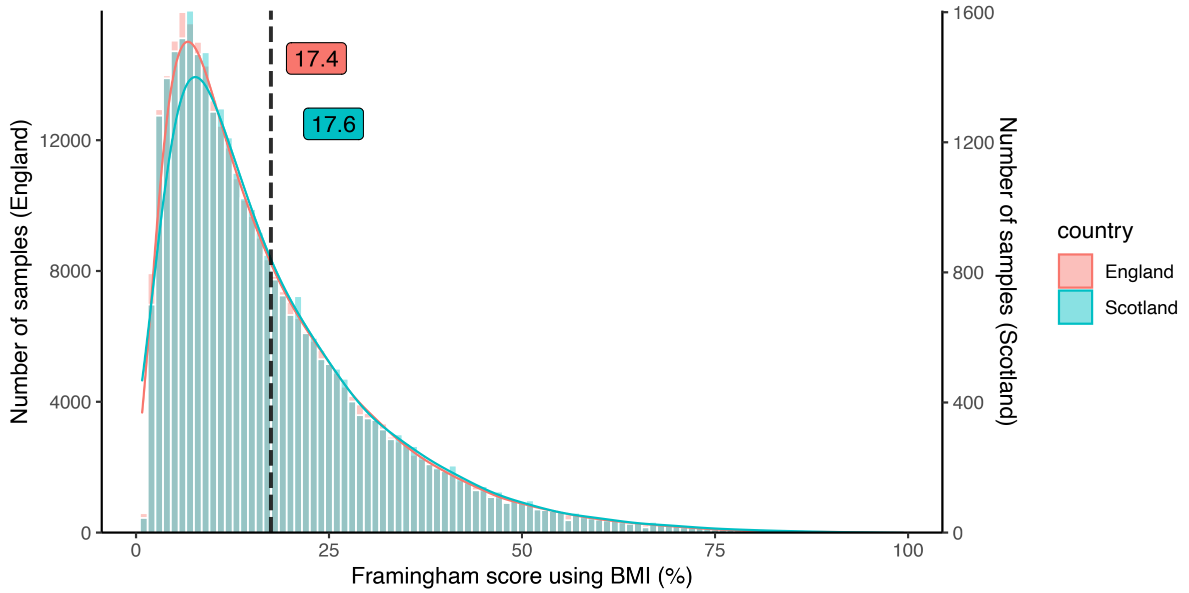

Supplementary Fig. 2 Histograms showing the distribution of the Framingham score using BMI for comparison population (born in Scotland) and reference population (born in England).

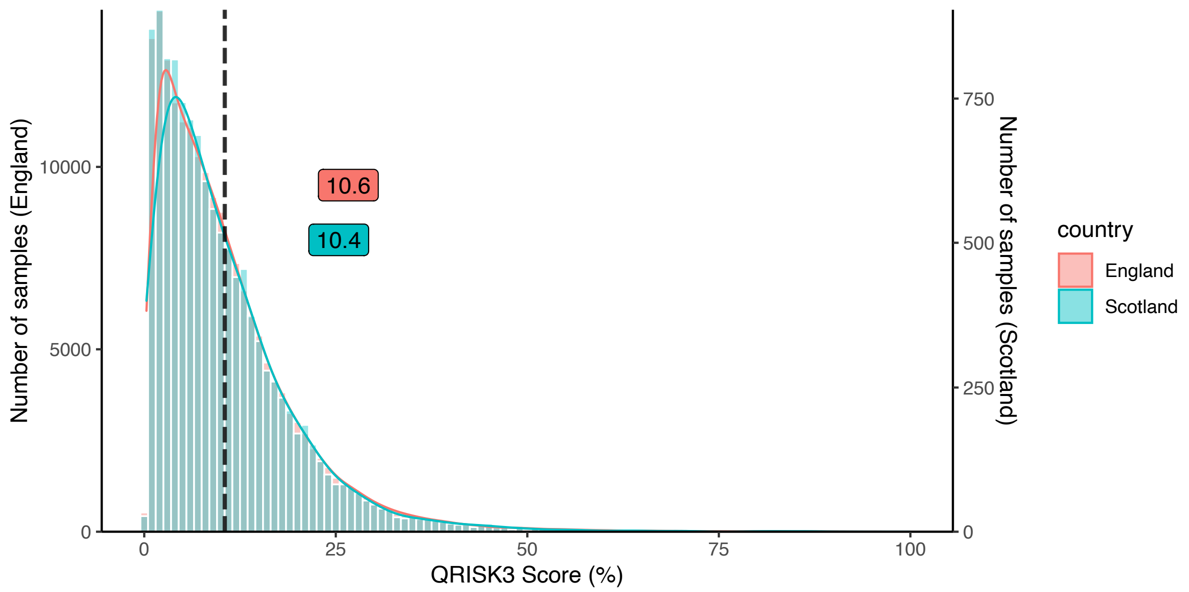

Supplementary Fig. 3 Histograms showing the distribution of the QRISK3 score for comparison population (born in Scotland) and reference population (born in England).

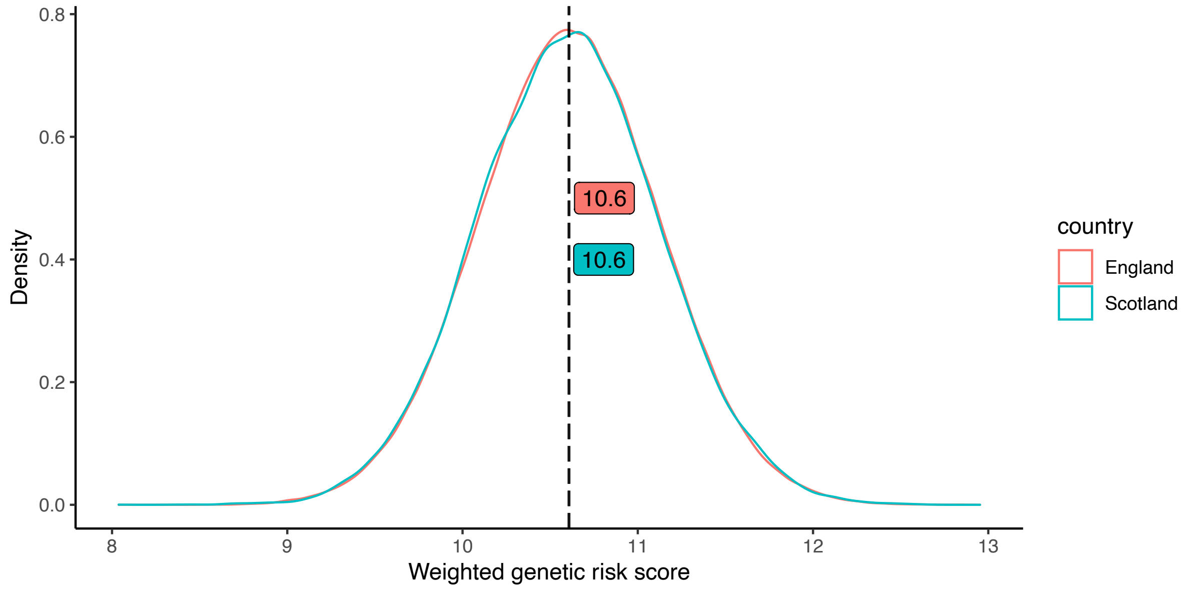

Supplementary Fig. 4 The density plot showing the distribution curves of weighted genetic risk score based on 163 CAD-associated SNPs for comparison population (born in Scotland) and reference population (born in England).

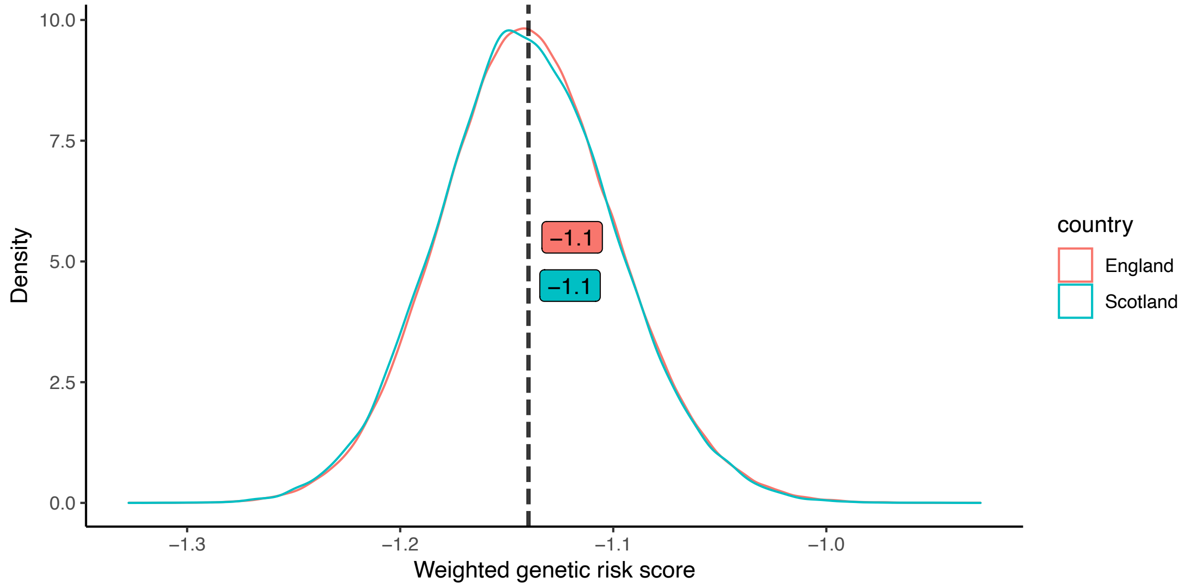

Supplementary Fig. 5 The density plot showing the distribution curves of weighted genetic risk score based on 6.6 million CAD-associated SNPs for comparison population (born in Scotland) and reference population (born in England).

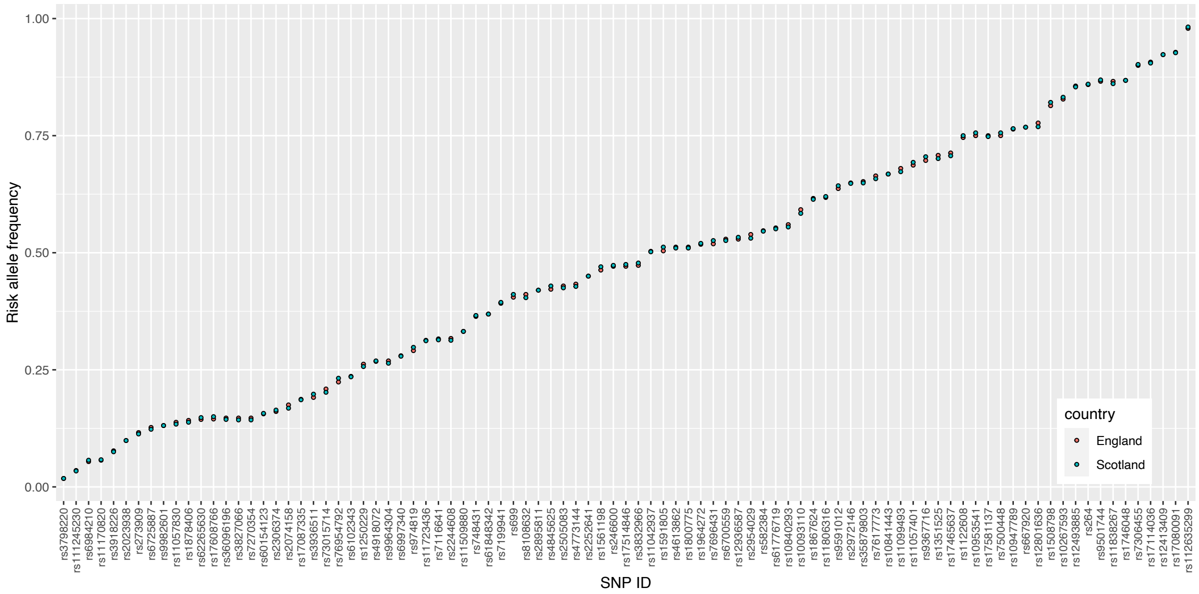

Supplementary Fig. 6 Dumbbell plot showing the risk allele frequency per SNP in England and Scotland. There was no significant difference between England and Scotland in risk allele frequency among 91 variants.

Supplementary Tables

Supplementary Table 1 Sample size with eligible genotype data and CAD prevalence in 4 countries of UK

Country of Birth	#Samples	CAD Prevalence (%)	P (Chi-squared test)
Scotland ***	39,130	9.06	<0.001
Wales **	21,567	8.27	0.004
Northern Ireland	2,990	8.06	0.506
England (reference)	379,173	7.73	/
	442,860	S>W>NI>E	/

* p < 0.05; ** p < 0.01; *** p < 0.001

Supplementary Table 2 Variables used when calculating the Framingham risk scores for Englander and Scottish individuals in UK Biobank at baseline

**Variable included in Framingham risk score**	**Measured by**	**UKBB Field ID**	**ICD Code**
**Biological Factors**			
Age	Baseline characteristic	21003	
Gender	Baseline characteristic	31	
Systolic blood pressure	Baseline characteristic	4080, 93	
HDL	Baseline characteristic	30760	
Cholesterol	Baseline characteristic	30690	
**Lifestyle**			
BMI	Baseline characteristic	21001	
Smoking	Self-report	20116	
**Diagnoses**			
Diabetes	Hospital episode statistics	41270, 41202, 41204	E10-E14
**Treatments**			
Antihypertensives	Nurses interview treatment data/ self-report	20003, 6177, 6153	

Supplementary Table 3 Variables used when calculating QRISK3 scores for Englander and Scottish individuals in UK Biobank at baseline

Variable included in QRISK3 algorithm	UKBB Field ID	ICD Code
**Biological Factors**		
Age	21003	
Systolic blood pressure	4080	
Systolic blood pressure variability	4080	
Total cholesterol: HDL ratio	30690, 30760	
CVD in first degree relative (<60 years)	20107, 20110, 20111	
**Lifestyle**		
Ethnicity	21000	
Townsend deprivation index	189	
BMI	21001	
Smoking	3456, 20116	
**Diagnoses**		
Arthritis	41270	M05
Type I Diabetes	41270	E10
Type II Diabetes	41270	E11-E14
Systemic lupus erythematosus	41270	M32.9
Atrial fibrillation	41270	I48
Chronic kidney disease	41270	N18.3-N18.5
Migraine	41270	G43
Severe mental illness	41270	F20, F23, F31, F32, F33
Erectile dysfunction	41270	N52
**Treatments**		
Antihypertensives	20003	
Corticosteroids	20003	
Second generation atypical Psychotics	20003	
Erectile dysfunction	20003	

Supplementary Table 4 Code list for antihypertensives in Data-Field 20003

Data Field 20003	Code
Antihypertensives	1140860332 1140860334 1140860336 1140860338 1140860340 1140860342 1140860348 1140860352 1140860356 1140860358 1140860362 1140860380 1140860382 1140860386 1140860390 1140860394 1140860396 1140860398 1140860402 1140860404 1140860406 1140860410 1140860418 1140860422 1140860426 1140860434 1140860454 1140860470 1140860478 1140860492 1140860498 1140860520 1140860532 1140860534 1140860544 1140860552 1140860558 1140860562 1140860564 1140860580 1140860590 1140860610 1140860628 1140860632 1140860638 1140860654 1140860658 1140860690 1140860696 1140860706 1140860714 1140860728 1140860736 1140860738 1140860750 1140860752 1140860758 1140860764 1140860776 1140860784 1140860790 1140860802 1140860806 1140860828 1140860830 1140860834 1140860836 1140860838 1140860840 1140860842 1140860846 1140860848 1140860862 1140860878 1140860882 1140860892 1140860904 1140860912 1140860918 1140860938 1140860942 1140860952 1140860954 1140860966 1140860972 1140860976 1140860982 1140860988 1140860994 1140861000 1140861002 1140861008 1140861010 1140861016 1140861022 1140861024 1140861034 1140861046 1140861068 1140861070 1140861088 1140861090 1140861106 1140861110 1140861114 1140861120 1140861128 1140861130 1140861136 1140861138 1140861166 1140861176 1140861190 1140861194 1140861202 1140861266 1140861268 1140861276 1140861282 1140861326 1140861384 1140864950 1140864952 1140866072 1140866074 1140866078 1140866084 1140866086 1140866090 1140866092 1140866094 1140866096 1140866102 1140866104 1140866108 1140866110 1140866116 1140866122 1140866128 1140866132 1140866136 1140866138 1140866140 1140866144 1140866146 1140866156 1140866158 1140866162 1140866164 1140866168 1140866182 1140866192 1140866194 1140866200 1140866202 1140866206 1140866210 1140866212 1140866220 1140866222 1140866226 1140866230 1140866232 1140866236 1140866244 1140866248 1140866262 1140866280 1140866282 1140866306 1140866308 1140866312 1140866318 1140866324 1140866328 1140866330 1140866332 1140866334 1140866340 1140866352 1140866354 1140866356 1140866360 1140866388 1140866390 1140866396 1140866400 1140866402 1140866404 1140866406 1140866408 1140866410 1140866412 1140866416 1140866418 1140866420 1140866422 1140866426 1140866438 1140866440 1140866442 1140866444 1140866446 1140866448 1140866450 1140866460 1140866466 1140866484 1140866506 1140866546 1140866554 1140866692 1140866704 1140866712 1140866724 1140866726 1140866738 1140866756 1140866758 1140866764 1140866766 1140866778 1140866782 1140866784 1140866798 1140866800 1140866802 1140866804

	1140875808 1140879758 1140879760 1140879762<br>1140879778 1140879782 1140879786 1140879794<br>1140879798 1140879802 1140879806 1140879810<br>1140879818 1140879822 1140879824 1140879826<br>1140879830 1140879834 1140879842 1140879854<br>1140879866 1140888510 1140888512 1140888552<br>1140888556 1140888560 1140888578 1140888582<br>1140888586 1140888646 1140888686 1140888760<br>1140888762 1140909368 1140911698 1140916356<br>1140916362 1140917428 1140923572 1140923712<br>1140923718 1140926778 1140926780 1141145658<br>1141145660 1141145668 1141151016 1141151018<br>1141151382 1141152600 1141152998 1141153006<br>1141153026 1141153032 1141153328 1141156754<br>1141156808 1141156836 1141156846 1141157252<br>1141157254 1141164148 1141164154 1141164276<br>1141164280 1141165470 1141165476 1141166006<br>1141167822 1141167832 1141171152 1141171336<br>1141171344 1141172682 1141172686 1141172698<br>1141173888 1141180592 1141180598 1141187788<br>1141187790 1141190160 1141192064 1141193282<br>1141193346 1141194794 1141194800 1141194804<br>1141194808 1141194810 1141201038 1141201040

Supplementary Table 5 Codes list for identification of CAD

**Hospital codes**
**ICD10 I21-I24,I25.2**
I21 I21 Acute myocardial infarction
I210 I21.0 Acute transmural myocardial infarction of anterior wall
I211 I21.1 Acute transmural myocardial infarction of inferior wall
I212 I21.2 Acute transmural myocardial infarction of other sites
I213 I21.3 Acute transmural myocardial infarction of unspecified site
I214 I21.4 Acute subendocardial myocardial infarction
I219 I21.9 Acute myocardial infarction, unspecified
I21X I21.X Presumed acute myicardial infaction (unconfirmed)
I22 I22 Subsequent myocardial infarction
I220 I22.0 Subsequent myocardial infarction of anterior wall
I221 I22.1 Subsequent myocardial infarction of inferior wall
I228 I22.8 Subsequent myocardial infarction of other sites
I229 I22.9 Subsequent myocardial infarction of unspecified site
I23 I23 Certain current complications following acute myocardial infarction
I23.0 Haemopericardium as current complication following acute myocardial I230 infarction
I23.1 Atrial septal defect as current complication following acute myocardial I231 infarction
I23.2 Ventricular septal defect as current complication following acute myocardial I232 infarction
I23.3 Rupture of cardiac wall without haemopericardium as current complication I233 following acute myocardial infarction
I23.4 Rupture of chordae tendineae as current complication following acute I234 myocardial infarction
I23.5 Rupture of papillary muscle as current complication following acute myocardial I235 infarction
I23.6 Thrombosis of atrium, auricular appendage and ventricle as current I236 complications following acute myocardial infarction
I238 I23.8 Other current complications following acute myocardial infarction

I24 I24 Other acute ischaemic heart diseases
I240 I24.0 Coronary thrombosis not resulting in myocardial infarction
I241 I24.1 Dressler's syndrome
I248 I24.8 Other forms of acute ischaemic heart disease
I249 I24.9 Acute ischaemic heart disease, unspecified
I252 I25.2 Old myocardial infarction
**ICD9 410-412**
410 410 Acute myocardial infarction
4109 4109 Acute myocardial infarction
411 411 Other acute and subacute forms of ischaemic heart disease
4119 4119 Other acute and subacute forms of ischaemic heart disease
412 412 Old myocardial infarction
4129 4129 Old myocardial infarction
**OPCS -4 K40- K46, K49, K50.1, K75**
K40 K40 Saphenous vein graft replacement of coronary artery
K401 K40.1 Saphenous vein graft replacement of one coronary artery
K402 K40.2 Saphenous vein graft replacement of two coronary arteries
K403 K40.3 Saphenous vein graft replacement of three coronary arteries
K404 K40.4 Saphenous vein graft replacement of four or more coronary arteries
K408 K40.8 Other specified saphenous vein graft replacement of coronary artery
K409 K40.9 Unspecified saphenous vein graft replacement of coronary artery
K41 K41 Other autograft replacement of coronary artery
K411 K41.1 Autograft replacement of one coronary artery NEC
K412 K41.2 Autograft replacement of two coronary arteries NEC
K413 K41.3 Autograft replacement of three coronary arteries NEC
K414 K41.4 Autograft replacement of four or more coronary arteries NEC
K418 K41.8 Other specified other autograft replacement of coronary artery
K419 K41.9 Unspecified other autograft replacement of coronary artery
K42 K42 Allograft replacement of coronary artery
K421 K42.1 Allograft replacement of one coronary artery
K422 K42.2 Allograft replacement of two coronary arteries
K423 K42.3 Allograft replacement of three coronary arteries
K424 K42.4 Allograft replacement of four or more coronary arteries
K428 K42.8 Other specified allograft replacement of coronary artery
K429 K42.9 Unspecified allograft replacement of coronary artery
K43 K43 Prosthetic replacement of coronary artery
K431 K43.1 Prosthetic replacement of one coronary artery
K432 K43.2 Prosthetic replacement of two coronary arteries
K433 K43.3 Prosthetic replacement of three coronary arteries
K434 K43.4 Prosthetic replacement of four or more coronary arteries
K438 K43.8 Other specified prosthetic replacement of coronary artery
K439 K43.9 Unspecified prosthetic replacement of coronary artery
K44 K44 Other replacement of coronary artery
K441 K44.1 Replacement of coronary arteries using multiple methods
K442 K44.2 Revision of replacement of coronary artery
K448 K44.8 Other specified other replacement of coronary artery
K449 K44.9 Unspecified other replacement of coronary artery
K45 K45 Connection of thoracic artery to coronary artery

K451 K45.1 Double anastomosis of mammary arteries to coronary arteries
K452 K45.2 Double anastomosis of thoracic arteries to coronary arteries NEC
K453 K45.3 Anastomosis of mammary artery to left anterior descending coronary artery
K454 K45.4 Anastomosis of mammary artery to coronary artery NEC
K455 K45.5 Anastomosis of thoracic artery to coronary artery NEC
K456 K45.6 Revision of connection of thoracic artery to coronary artery
K458 K45.8 Other specified connection of thoracic artery to coronary artery
K459 K45.9 Unspecified connection of thoracic artery to coronary artery
K46 K46 Other bypass of coronary artery
K461 K46.1 Double implantation of mammary arteries into heart
K462 K46.2 Double implantation of thoracic arteries into heart NEC
K463 K46.3 Implantation of mammary artery into heart NEC
K464 K46.4 Implantation of thoracic artery into heart NEC
K465 K46.5 Revision of implantation of thoracic artery into heart
K468 K46.8 Other specified other bypass of coronary artery
K469 K46.9 Unspecified other bypass of coronary artery
K49 K49 Transluminal balloon angioplasty of coronary artery
K491 K49.1 Percutaneous transluminal balloon angioplasty of one coronary artery
K492 K49.2 Percutaneous transluminal balloon angioplasty of multiple coronary arteries
K49.3 Percutaneous transluminal balloon angioplasty of bypass graft of coronary K493 artery
K494 K49.4 Percutaneous transluminal cutting balloon angioplasty of coronary artery
K498 K49.8 Other specified transluminal balloon angioplasty of coronary artery
K499 K49.9 Unspecified transluminal balloon angioplasty of coronary artery
K50 K50 Other therapeutic transluminal operations on coronary artery
K501 K50.1 Percutaneous transluminal laser coronary angioplasty
K502 K50.2 Percutaneous transluminal coronary thrombolysis using streptokinase
K50.3 Percutaneous transluminal injection of therapeutic substance into coronary K503 artery NEC
K504 K50.4 Percutaneous transluminal atherectomy of coronary artery
K508 K50.8 Other specified other therapeutic transluminal operations on coronary artery
K509 K50.9 Unspecified other therapeutic transluminal operations on coronary artery
K75 Percutaneous transluminal balloon angioplasty and insertion of stent into K75 coronary artery
K75.1 Percutaneous transluminal balloon angioplasty and insertion of 1-2 drug- K751 eluting stents into coronary artery
K75.2 Percutaneous transluminal balloon angioplasty and insertion of 3 or more K752 drug-eluting stents into coronary artery
K75.3 Percutaneous transluminal balloon angioplasty and insertion of 1-2 stents K753 into coronary artery
K75.4 Percutaneous transluminal balloon angioplasty and insertion of 3 or more K754 stents into coronary artery NEC
K75.8 Other specified percutaneous transluminal balloon angioplasty and insertion K758 of stent into coronary artery
K75.9 Unspecified percutaneous transluminal balloon angioplasty and insertion of K759 stent into coronary artery
**2. Self-reported**
1075 heart attack/myocardial infarction
1070 coronary angioplasty (ptca) +/- stent
1095 coronary artery bypass grafts (cabg)
1523 triple heart bypass

Supplementary Table 6 Sample size with both eligible genotype data and variables for Framingham score and CAD prevalence in 4 countries of UK

Country of Birth	#Samples	CAD Prevalence (%)	P (Chi-squared test)
Scotland ***	31,963	8.98	<0.001
Wales**	18,724	8.30	0.002
Northern Ireland	2,501	8.36	0.205
England (reference)	317,889	7.68	/
	371,077	S>NI>W>E	/

* p < 0.05; ** p < 0.01; *** p < 0.001

Supplementary Table 7 Comparison of the risk allele frequencies across 163 CAD-associated SNPs between England and Scotland

Position	SNP	Risk allele	OR	England_ RAF	Scotland_ RAF	Delta_ RAF	P (Chi-squared)	Gene(s) at locus
chr1:38,461,319	rs61776719	A	1.04	0.553	0.551	-0.002	1.00E+00	FHL3, UTP11, SF3A3, MANEAL, INPP5B
chr1:55,496,039	rs11206510	T	1.08	0.811	0.819	0.008	1.53E-04	PCSK9
chr1:56,962,821	rs17114036	A	1.17	0.907	0.905	-0.002	1.00E+00	PPAP2B
chr1:109,822,166	rs599839	A	1.11	0.77	0.759	-0.011	2.35E-08	SORT1, PSCR1, CELSR2
chr1:115,753,482	rs11806316	G	1.04	0.618	0.62	0.002	1.00E+00	NGF, CASQ2
chr1:151,762,308	rs11810571	G	1.07	0.842	0.828	-0.014	7.44E-17	TDRKH, RP11-98D18.9
chr1:200,646,073	rs6700559	C	1.04	0.529	0.526	-0.003	1.00E+00	DDX59, CAMSAP2, KIF14
chr1:222,823,529	rs17465637	C	1.14	0.713	0.707	-0.007	7.37E-02	MIA3, AIDA, C1orf58
chr2:21,286,057	rs515135	C	1.07	0.817	0.827	0.01	1.66E-08	APOB
chr2:45,896,437	rs582384	A	1.03	0.547	0.546	-0.001	1.00E+00	PRKCE, TMEM247
chr2:164,957,251	rs12999907	A	1.06	0.825	0.817	-0.008	2.32E-04	FIGN
chr2:188,196,469	rs840616	C	1.04	0.66	0.672	0.013	1.39E-08	CALCRL, TFPI
chr2:227,100,698	rs2972146	T	1.07	0.649	0.648	-0.001	1.00E+00	LOC646736, IRS1, MIR5702
chr2:238,223,955	rs11677932	G	1.03	0.68	0.697	0.017	6.89E-16	COL6A3
chr3:48,193,515	rs7617773	T	1.04	0.664	0.658	-0.005	1.00E+00	CDC25A, SPINK8, MAP4, ZNF589
chr3:49,448,566	rs7623687	A	1.07	0.859	0.849	-0.01	4.55E-09	RHOA, AMT, TCTA, CDHRA, KLHDC8B, and others
chr3:132,257,961	rs10512861	G	1.04	0.868	0.861	-0.007	4.67E-05	DNAJC13, NPHP3, ACAD11, UBA5
chr3:136,069,472	rs667920	T	1.05	0.768	0.768	0.001	1.00E+00	STAG1, MSL2, NCK1, PPP2R3A
chr3:153,839,866	rs12493885	C	1.07	0.856	0.854	-0.002	1.00E+00	ARHGEF26
chr4:82,587,050	rs11099493	A	1.04	0.68	0.673	-0.008	1.16E-02	HNRNPD, RASGEF1B
chr4:146,782,837	rs35879803	C	1.05	0.652	0.649	-0.003	1.00E+00	ZNF827
chr4:156,635,309	rs7692387	G	1.08	0.817	0.809	-0.008	1.19E-04	GUCY1A1*
chr4:169,687,725	rs7696431	T	1.04	0.519	0.526	0.007	1.99E-01	PALLD, DDX60L
chr5:9,556,694	rs1508798	T	1.05	0.814	0.821	0.007	1.05E-03	SEMA5A, TAS2R1
chr6:1,617,143	rs9501744	C	1.05	0.866	0.869	0.003	1.00E+00	FOXC1
chr6:12,927,544	rs12526453	C	1.1	0.668	0.681	0.013	8.32E-09	PHACTR1, EDN1
chr6:31,888,367	rs3130683	T	1.09	0.856	0.839	-0.018	1.08E-31	C2, C4A, and others

chr6:35,034,800	rs17609940	G	1.07	0.784	0.765	-0.019	7.53E-27	ANKS1A, UHRF1BP1
chr6:39,174,922	rs10947789	T	1.07	0.764	0.765	0	1.00E+00	KCNK5
chr6:43,758,873	rs6905288	A	1.05	0.567	0.584	0.017	2.20E-14	VEGFA, MRPL14, TMEM63B
chr6:57,160,572	rs9367716	G	1.04	0.697	0.705	0.008	4.27E-03	PRIM2, RAB23, DST, BEND6
chr6:82,612,271	rs4613862	A	1.03	0.512	0.51	-0.002	1.00E+00	FAM46A
chr6:134,214,525	rs12190287	C	1.08	0.629	0.619	-0.01	4.90E-05	TCF21, TARID (EYA4,àíAS1)
chr6:150,997,401	rs17080091	C	1.05	0.928	0.927	-0.001	1.00E+00	PLEKHG1, IYD
chr6:161,143,608	rs4252120	T	1.07	0.708	0.722	0.014	2.99E-12	PLG, LPAL2
chr7:1,937,261	rs10267593	G	1.04	0.828	0.832	0.004	1.00E+00	MAD1L1
chr7:6,486,067	rs7797644	C	1.04	0.767	0.75	-0.016	5.84E-18	DAGLB, RAC1, FAM220A, KDELR2
chr7:45,077,978	rs2107732	G	1.06	0.91	0.897	-0.013	3.66E-26	CCM2, MYO1G
chr7:107,244,545	rs10953541	C	1.08	0.75	0.756	0.006	1.77E-01	BCAP29, GPR22
chr7:129,663,496	rs11556924	C	1.09	0.615	0.605	-0.01	4.21E-05	ZC3HC1, KLHDC10
chr7:139,757,136	rs10237377	G	1.05	0.659	0.675	0.016	8.27E-14	PARP12, TBXAS1
chr8:19,813,180	rs264	G	1.11	0.859	0.86	0.002	1.00E+00	LPL
chr8:106,565,414	rs10093110	G	1.03	0.592	0.584	-0.008	1.82E-02	ZFPM2
chr8:126,490,972	rs2954029	A	1.06	0.539	0.531	-0.007	6.77E-02	TRIB1
chr10:44,775,824	rs1746048	C	1.09	0.868	0.868	0	1.00E+00	CXCL12
chr10:82,251,514	rs17680741	T	1.05	0.713	0.724	0.011	7.86E-07	TSPAN14, MAT1A, FAM213A
chr10:104,719,096	rs12413409	G	1.12	0.923	0.923	0	1.00E+00	CYP17A1, CNNM2, NT5C2
chr11:9,751,196	rs10840293	A	1.06	0.56	0.555	-0.005	1.00E+00	SWAP70
chr11:10,745,394	rs11042937	T	1.03	0.503	0.502	-0.002	1.00E+00	MRVI1, CTR9
chr11:13,301,548	rs1351525	T	1.05	0.708	0.701	-0.007	5.35E-02	ARNTL
chr11:65,391,317	rs12801636	G	1.05	0.777	0.769	-0.007	3.52E-03	PCNX3, POLA2, RELA, SIPA1, and others
chr12:7,175,872	rs11838267	T	1.05	0.866	0.861	-0.004	3.57E-01	C1S
chr12:20,220,033	rs10841443	G	1.06	0.668	0.668	0	1.00E+00	RP11-664H17.1
chr12:95,355,541	rs7306455	G	1.05	0.9	0.902	0.002	1.00E+00	NDUFA12, FGD6
chr12:124,427,306	rs11057401	T	1.08	0.687	0.693	0.005	7.16E-01	CCDC92
chr13:33,058,333	rs9591012	G	1.04	0.637	0.643	0.005	1.00E+00	N4BP2L2, PDS5B
chr14:94,838,142	rs112635299	G	1.13	0.979	0.982	0.002	5.03E-03	SERPINA2, SERPINA1
chr15:65,024,204	rs6494488	A	1.05	0.852	0.859	0.007	6.04E-04	OAZ2, RBPMS2, TRIP4, and others
chr15:67,455,630	rs56062135	C	1.07	0.764	0.775	0.01	6.31E-07	SMAD3
chr15:79,089,111	rs3825807	A	1.08	0.555	0.545	-0.01	5.71E-04	ADAMTS7
chr15:89,574,218	rs8042271	G	1.1	0.961	0.968	0.007	7.58E-15	MFGE8, RP11-326A19.4, ABHD2
chr15:96,146,414	rs17581137	A	1.04	0.75	0.748	-0.002	1.00E+00	gene desert
chr16:56,961,074	rs1800775	C	1.03	0.512	0.51	-0.003	1.00E+00	CETP
chr16:75,387,533	rs3851738	C	1.07	0.593	0.605	0.011	4.79E-06	CFDP1, BCAR1
chr16:83,045,790	rs7500448	A	1.07	0.75	0.756	0.005	4.79E-01	CDH13
chr17:17,543,722	rs12936587	G	1.07	0.529	0.533	0.004	1.00E+00	Ral1, PEMT, RASD1, SMCR3, TOM1L2
chr17:46,988,597	rs46522	T	1.06	0.529	0.516	-0.013	4.45E-08	UBE2Z, GIP, ATP5G1
chr17:62,387,091	rs1867624	T	1.04	0.616	0.614	-0.003	1.00E+00	PECAM1, DDX5, TEX2
chr19:8,429,323	rs116843064	G	1.14	0.981	0.978	-0.003	2.63E-04	ANGTPL4

chr19:11,163,601	rs1122608	G	1.14	0.746	0.75	0.004	1.00E+00	LDLR, SMARCA4
chr19:32,882,020	rs12976411	A	1.33	0.961	0.956	-0.005	6.34E-08	ZNF507, LOC400684
chr19:46,190,268	rs1964272	G	1.04	0.518	0.52	0.002	1.00E+00	SNRPD2, GIPR
chr20:33,764,554	rs867186	A	1.07	0.911	0.92	0.009	3.00E-12	PROCR, ASIP, NCOA6, ITGB4BP/EIF6 and others
chr22:24,262,640	rs180803	G	1.2	0.988	0.991	0.004	3.83E-13	ADORA2A
chr1:2,252,205	rs36096196	T	1.05	0.147	0.144	-0.004	1.00E+00	MORN1, SKI
chr1:3,325,912	rs2493298	A	1.06	0.139	0.13	-0.009	3.45E-08	PRDM16, PEX10, PLCH2, RER1
chr1:154,422,067	rs4845625	T	1.06	0.422	0.429	0.007	8.48E-02	IL6R, AQP10, ATP8B2, CHTOP, UBAP2L
chr1:169,094,459	rs1892094	C	1.04	0.478	0.46	-0.018	2.70E-16	ATP1B1, BLZF1, CCDC181, F5, NME7, SELP, SLC19A2
chr1:201,872,264	rs2820315	T	1.05	0.316	0.33	0.013	7.42E-10	LMOD1, IPO9, NAV1, SHISA4, TIMM17A
chr1:210,468,999	rs60154123	T	1.05	0.156	0.157	0.001	1.00E+00	HHAT, SERTAD4, DIEXF
chr1:230,845,794	rs699	G	1.04	0.405	0.411	0.006	7.44E-01	AGT, CAPN9, GNPAT
chr2:44,073,881	rs6544713	T	1.06	0.32	0.34	0.02	1.59E-23	ABCG5, ABCG8
chr2:85,809,989	rs1561198	T	1.06	0.463	0.47	0.007	2.06E-01	VAMP5, VAMP8, GGCX
chr2:145,801,461	rs2252641	C	1.06	0.45	0.45	0	1.00E+00	ZEB2, TEX41
chr2:203,745,885	rs6725887	C	1.14	0.127	0.123	-0.004	5.33E-01	WDR12, CARF, FAM117B, ICA1L, NBEAL1
chr2:216,304,384	rs1250229	T	1.07	0.262	0.257	-0.006	3.70E-01	FN1, ATIC, LOC102724849, ABCA12, LINC00607
chr2:218,683,154	rs2571445	A	1.04	0.393	0.404	0.011	1.17E-05	TNS1, CXCR2, RUFY4
chr2:233,633,460	rs1801251	A	1.05	0.362	0.383	0.021	2.36E-24	KCNJ13, GIGYF2
chr3:14,928,077	rs748431	G	1.04	0.364	0.366	0.002	1.00E+00	FGD5
chr3:46,688,562	rs7633770	A	1.03	0.423	0.437	0.014	2.15E-09	ALS2CL, RTP3
chr3:124,475,201	rs62265630	G	1.08	0.144	0.148	0.003	1.00E+00	UMPS, ITGB5
chr3:138,119,952	rs2306374	C	1.12	0.161	0.164	0.003	1.00E+00	MRAS, CEP70
chr3:172,115,902	rs12897	G	1.04	0.373	0.356	-0.017	6.44E-15	FNDC3B
chr4:3,449,652	rs16844401	A	1.07	0.066	0.059	-0.007	3.96E-09	HGFAC, RGS12, MSANTD1
chr4:57,838,583	rs17087335	T	1.06	0.187	0.186	-0.001	1.00E+00	REST, NOA1
chr4:77,416,627	rs12500824	A	1.04	0.353	0.364	0.011	8.36E-06	SHROOM3, SEPT11, FAM47E, STBD1
chr4:120,901,336	rs11723436	G	1.05	0.313	0.312	-0.001	1.00E+00	MAD2L1, PDE5A
chr4:147,472,512	rs1878406	T	1.1	0.142	0.138	-0.004	7.02E-01	EDNRA
chr5:55,860,781	rs3936511	G	1.04	0.191	0.198	0.007	1.42E-03	MAP3K1, MIER3
chr5:121,413,208	rs1800449	T	1.09	0.169	0.18	0.011	8.47E-11	LOX
chr5:131,667,353	rs273909	G	1.07	0.116	0.113	-0.003	1.00E+00	SLC22A4
chr5:131,867,702	rs2706399	G	1.07	0.506	0.519	0.013	2.47E-08	IL5, RAD50
chr5:142,516,897	rs246600	T	1.05	0.471	0.473	0.002	1.00E+00	ARHGAP26
chr6:36,638,636	rs1321309	A	1.03	0.502	0.513	0.011	1.99E-05	CDKN1A, PI16
chr6:126,717,064	rs1591805	A	1.04	0.504	0.512	0.009	5.66E-03	CENPW
chr6:160,961,137	rs3798220	C	1.51	0.018	0.018	0	1.00E+00	LPA, SLC22A3, LPAL2
chr7:12,261,911	rs11509880	A	1.04	0.332	0.332	0	1.00E+00	TMEM106B, THSD7A
chr7:19,036,775	rs2023938	C	1.08	0.099	0.099	-0.001	1.00E+00	HDAC9
chr7:117,332,914	rs975722	G	1.03	0.386	0.376	-0.01	1.11E-04	CTTNBP2, CFTR, ASZ1
chr7:150,690,176	rs3918226	T	1.14	0.077	0.075	-0.002	1.00E+00	NOS3
chr8:18,286,997	rs6997340	T	1.04	0.28	0.279	-0.001	1.00E+00	NAT2

chr9:110,517,794	rs944172	C	1.04	0.281	0.293	0.012	4.83E-09	KLF4
chr9:113,169,775	rs111245230	C	1.14	0.035	0.034	-0.001	1.00E+00	SVEP1
chr9:124,420,173	rs885150	C	1.03	0.26	0.245	-0.016	6.33E-16	DAB2IP
chr9:136,154,168	rs579459	C	1.1	0.21	0.176	-0.033	9.87E-86	ABO, SURF6, GBGT1
chr10:12,303,813	rs61848342	C	1.04	0.369	0.369	0	1.00E+00	CDC123, NUDT5, OPTN
chr10:30,335,122	rs2505083	C	1.07	0.429	0.425	-0.004	1.00E+00	KIAA1462
chr10:91,002,927	rs1412444	T	1.09	0.337	0.357	0.02	4.45E-22	LIPA
chr10:105,693,644	rs4918072	A	1.04	0.268	0.269	0	1.00E+00	STN1, SH3PXD2A
chr10:124,237,612	rs4752700	G	1.03	0.435	0.421	-0.014	1.19E-09	HTRA1, PLEKHA1
chr11:5,701,074	rs11601507	A	1.09	0.068	0.075	0.007	9.27E-09	TRIM5, TRIM22, TRIM6, OR52N1, OR52B6
chr11:43,696,917	rs7116641	G	1.03	0.316	0.314	-0.003	1.00E+00	HSD17B12
chr11:75,274,150	rs590121	T	1.05	0.289	0.3	0.011	1.20E-06	SERPINH1
chr11:100,624,599	rs7947761	G	1.04	0.292	0.314	0.022	7.39E-29	ARHGAP42
chr11:103,660,567	rs974819	T	1.07	0.291	0.298	0.007	1.47E-02	PDGFD
chr12:57,527,283	rs11172113	C	1.06	0.411	0.421	0.01	4.20E-04	LRP1, STAT6
chr12:111,884,608	rs3184504	T	1.07	0.478	0.495	0.017	4.80E-14	SH2B3, FLJ21127, ATXN2, and others
chr12:118,265,441	rs11830157	G	1.12	0.401	0.377	-0.024	1.11E-29	KSR2
chr12:121,416,988	rs2244608	G	1.06	0.317	0.313	-0.004	1.00E+00	HNF1A, OASL, C12orf43, and others
chr12:125,307,053	rs11057830	A	1.07	0.138	0.134	-0.005	1.76E-01	SCARB1
chr13:28,973,621	rs9319428	A	1.06	0.296	0.287	-0.009	3.48E-04	FLT1
chr13:110,960,712	rs4773144	G	1.07	0.433	0.428	-0.005	1.00E+00	COL4A1, COL4A2
chr13:113,631,780	rs1317507	A	1.04	0.256	0.248	-0.008	6.45E-04	MCF2L, PCID2, CUL4A
chr14:58,794,001	rs2145598	G	1.03	0.425	0.415	-0.01	1.80E-04	ARID4A, PSMA3
chr14:75,147,552	rs3832966	ACCC G	1.05	0.473	0.478	0.005	1.00E+00	TMED10, ZC2HC1C, RPS6KL1, NEK9, EIF2B2e, ACYP1
chr14:100,133,942	rs2895811	C	1.07	0.42	0.42	0	1.00E+00	HHIPL1, YY1
chr15:91,416,550	rs17514846	A	1.07	0.471	0.475	0.004	1.00E+00	FURIN, FES
chr16:72,096,666	rs1050362	A	1.04	0.352	0.342	-0.01	5.26E-05	DHX38, HP, DHODH
chr16:81,906,423	rs7199941	A	1.04	0.392	0.394	0.001	1.00E+00	PLCG2, CENPN
chr17:27,941,886	rs13723	G	1.04	0.493	0.503	0.01	3.16E-04	CORO6, BLMH, ANKRD13B, GIT1, SSH2, EFCAB5
chr17:30,033,514	rs76954792	T	1.04	0.224	0.232	0.008	1.82E-03	COPRS, RAB11FIP4
chr17:40,257,163	rs2074158	C	1.05	0.175	0.168	-0.007	3.47E-03	DHX58, KAT2A, RAB5, NKIRAS2, DNAJC7, KCNH4, HCRT, GHDC
chr17:45,013,271	rs17608766	C	1.07	0.145	0.15	0.004	4.53E-01	GOSR2, MYL4, ARL17A, and others
chr17:59,013,488	rs7212798	C	1.08	0.152	0.143	-0.009	5.12E-08	BCAS3
chr18:47,229,717	rs9964304	C	1.04	0.269	0.264	-0.005	7.93E-01	ACAA2, RPL17
chr18:57,838,401	rs663129	A	1.06	0.235	0.225	-0.01	6.70E-07	PMAIP1, MC4R
chr19:45,395,619	rs2075650	G	1.14	0.146	0.153	0.007	1.63E-04	APOE, APOC1, TOMM40, PVRL2, COTL1
chr20:39,924,279	rs6102343	A	1.04	0.236	0.235	-0.002	1.00E+00	ZHX3, PLCG1, TOP1
chr20:44,586,023	rs3827066	T	1.04	0.147	0.143	-0.004	1.00E+00	PCIF1, ZNF335, NEURL2, PLTP
chr20:44,607,661	rs7270354	A	1.06	0.147	0.143	-0.004	1.00E+00	MMP9
chr20:57,714,025	rs260020	T	1.04	0.125	0.114	-0.011	3.30E-13	ZNF831
chr21:30,533,076	rs2832227	G	1.04	0.162	0.148	-0.014	3.27E-18	MAP3K7CL, BACH1
chr21:35,599,128	rs9982601	T	1.18	0.131	0.131	0	1.00E+00	MRPS6, SLC5A3, KCNE2

chr3:156,852,592	rs4266144	G	1.03	0.301	0.286	-0.014	1.61E-11	CCNL1, TIPARP
chr4:81,181,072	rs10857147	T	1.06	0.288	0.279	-0.009	2.46E-04	PRDM8, FGF5
chr4:96,117,371	rs3775058	A	1.04	0.211	0.222	0.011	2.52E-08	UNC5C
chr8:22,033,615	rs6984210	G	1.08	0.054	0.057	0.003	3.81E-01	BMP1, SFTPC, DMTN, PHYHIP, DOK2, XPO7
chr9:22,125,503	rs1333049	C	1.29	0.479	0.496	0.017	2.50E-14	ANRIL, CDKN2B-AS
chr11:116,648,917	rs964184	G	1.13	0.135	0.123	-0.011	7.66E-14	APOA1-C3-A4-A5
chr12:54,513,915	rs11170820	G	1.1	0.057	0.058	0.001	1.00E+00	HOXC4
chr17:2,126,504	rs216172	C	1.07	0.352	0.338	-0.014	7.92E-11	SMG6, SRR
chr19:17,855,763	rs73015714	G	1.06	0.209	0.202	-0.006	5.17E-02	FCHO1, COLGALT1
chr19:41,854,534	rs8108632	T	1.05	0.411	0.404	-0.007	1.92E-01	HNRNPUL1, CCDC97, TGFB1, B9D2

RAF, risk allele frequency
P-value was adjusted by Bonferroni correction.

Supplementary Table 8 Characteristics of individuals aged 39-74 in two sub-cohorts from UK Biobank for calculating QRISK3 score

	England		Scotland	
	Women (N=108024)	Men (N=79881)	Women (N=6959)	Men (N=4914)
**Age (years)**				
Mean (SD)	57 (± 7.9)	58 (± 7.8)	57 (± 7.8)	58 (± 7.9)
**Townsend deprivation scores**				
Mean (SD)	-1.7 (± 2.8)	-1.7 (± 2.9)	-1.2 (± 3.2)	-1.3 (± 3.4)
**BMI**				
Mean (SD)	27 (± 5.2)	28 (± 4.3)	27 (± 5.2)	28 (± 4.2)
**Cholesterol/HDL ratio**				
Mean (SD)	3.8 (± 1.0)	4.4 (± 1.1)	3.8 (± 1.0)	4.4 (± 1.1)
**Systolic blood pressure (mmHg)**				
Mean (SD)	140 (± 19)	140 (± 17)	140 (± 19)	140 (± 18)
**Ethiniciy**				
White or not stated	106585 (98.67 %)	79154 (99.09 %)	6936 (99.67 %)	4900 (99.72 %)
Indian	137 (0.13 %)	93 (0.12 %)	2 (0.03 %)	1 (0.02 %)
Pakistani	42 (0.04 %)	36 (0.05 %)	3 (0.04 %)	1 (0.02 %)
Bangladeshi	2 (0.00 %)	1 (0.00 %)	0 (0.00 %)	0 (0.00 %)
Other Asian	19 (0.02 %)	16 (0.02 %)	0 (0.00 %)	0 (0.00 %)
Black Caribbean	467 (0.43 %)	182 (0.23 %)	1 (0.01 %)	0 (0.00 %)
Black African	77 (0.07 %)	37 (0.05 %)	0 (0.00 %)	0 (0.00 %)
Chinese	25 (0.02 %)	21 (0.03 %)	0 (0.00 %)	0 (0.00 %)
Other ethnic group	670 (0.62 %)	341 (0.43 %)	17 (0.24 %)	12 (0.24 %)
**Smoke status**				
non-smoker	68319 (63.24 %)	40730 (50.99 %)	4335 (62.29 %)	2639 (53.70 %)
ex-smoker	38788 (35.91 %)	38170 (47.78 %)	2555 (36.72 %)	2200 (44.77 %)
light smoker (less than 10)	267 (0.25 %)	172 (0.22 %)	19 (0.27 %)	14 (0.28 %)
moderate smoker (10 to 19)	362 (0.34 %)	390 (0.49 %)	21 (0.30 %)	23 (0.47 %)
heavy smoker (20 or over)	288 (0.27 %)	419 (0.52 %)	29 (0.42 %)	38 (0.77 %)
**Positive family history of CVD**				
Yes	14776 (13.68 %)	10736 (13.44 %)	1019 (14.64 %)	713 (14.51 %)
No	93248 (86.32 %)	69145 (86.56 %)	5940 (85.36 %)	4201 (85.49 %)
**Type 1 diabetes**				
Yes	789 (0.73 %)	1061 (1.33 %)	36 (0.52 %)	52 (1.06 %)
No	107235 (99.27 %)	78820 (98.67 %)	6923 (99.48 %)	4862 (98.94 %)
**Type 2 diabetes**				
Yes	5135 (4.75 %)	7790 (9.75 %)	280 (4.02 %)	396 (8.06 %)
No	102889 (95.25 %)	72091 (90.25 %)	6679 (95.98 %)	4518 (91.94 %)
**Treated hypertension**				
Yes	13624 (12.61 %)	13633 (17.07 %)	812 (11.67 %)	809 (16.46 %)
No	94400 (87.39 %)	66248 (82.93 %)	6147 (88.33 %)	4105 (83.54 %)
**Rheumatoid arthritis**				
Yes	326 (0.30 %)	105 (0.13 %)	21 (0.30 %)	3 (0.06 %)
No	107698 (99.70 %)	79776 (99.87 %)	6938 (99.70 %)	4911 (99.94 %)
**Atrial fibrillation**				
Yes	3535 (3.27 %)	6698 (8.38 %)	217 (3.12 %)	345 (7.02 %)
No	104489 (96.73 %)	73183 (91.62 %)	6742 (96.88 %)	4569 (92.98 %)
**Chronic kidney disease (stage 3, 4, or 5)**				
Yes	1199 (1.11 %)	1118 (1.40 %)	46 (0.66 %)	41 (0.83 %)
No	106825 (98.89 %)	78763 (98.60 %)	6913 (99.34 %)	4873 (99.17 %)
**Migraine**				
Yes	1553 (1.44 %)	441 (0.55 %)	65 (0.93 %)	21 (0.43 %)
No	106471 (98.56 %)	79440 (99.45 %)	6894 (99.07 %)	4893 (99.57 %)
**Corticosteroid use**				
Yes	2971 (2.75 %)	2581 (3.23 %)	181 (2.60 %)	160 (3.26 %)
No	105053 (97.25 %)	77300 (96.77 %)	6778 (97.40 %)	4754 (96.74 %)
**Systemic lupus erythematosus**				
Yes	208 (0.19 %)	20 (0.03 %)	10 (0.14 %)	4 (0.08 %)
No	107816 (99.81 %)	79861 (99.97 %)	6949 (99.86 %)	4910 (99.92 %)
**Atypical antipsychotic use**				
Yes	51 (0.05 %)	73 (0.09 %)	4 (0.06 %)	6 (0.12 %)
No	107973 (99.95 %)	79808 (99.91 %)	6955 (99.94 %)	4908 (99.88 %)
**Severe mental illness**				
Yes	5716 (5.29 %)	3079 (3.85 %)	178 (2.56 %)	107 (2.18 %)
No	102308 (94.71 %)	76802 (96.15 %)	6781 (97.44 %)	4807 (97.82 %)
**Erectile dysfunction diagnosis or treatment**				
Yes	0 (0.00 %)	277 (0.35 %)	0 (0.00 %)	13 (0.26 %)
No	108024 (100.00 %)	79604 (99.65 %)	6959 (100.00 %)	4901 (99.74 %)

Supplementary Table 9 Statistics for the Framingham score, QRISK3 score and genetic risk score in populations

	**England (N=317,889)**	**Scotland (N=319,63)**	**P-value**
**Framingham score using BMI****			0.0077
Mean (SD)	17.4 (13.5)	17.6 (13.7)	
Median [Min, Max]	13.4 [0.8, 99.4]	13.6 [1.0, 96.5]	
	**England (N= 187,905)**	**Scotland (N=11,873)**	**P-value**
**QRISK3 score***			0.0122
Mean (SD)	10.6 (9.3)	10.4 (9.0)	
Median [Min, Max]	8.3 [0.3, 99.9]	8.0 [0.3, 92.6]	
	**England (N=289,039)**	**Scotland (N=29,344)**	**P-value**
**wGRS (6.6 million variants)*****			< 0.001
Mean (SD)	-1.1 (0.0)	-1.1 (0.0)	
Median [Min, Max]	-1.1 [-1.3, -0.9]	-1.1 [-1.3, -0.9]	

SD: standard deviation

** p < 0.05; ** p < 0.01; *** p < 0.001;*

Supplementary Table 10 Script written for calculating the Framingham risk score using lipids and BMI

```
# calculating the Framingham risk score using lipids
frisk_lipid_calc <- function(gender, Age, HDL_cholesterol, Total_cholesterol,
                    Systolic_blood_pressure, is_sbp_under_treatment, Cigarette_smoker,
Diabetes_present){
  if(gender=="M" || gender=="m"){
    if(is_sbp_under_treatment==1){
      Risk_Factors = (log(Age) * 3.06117) + (log(Total_cholesterol) * 1.12370) -
(log(HDL_cholesterol) * 0.93263) +
        (log(Systolic_blood_pressure) * 1.99881) + Cigarette_smoker*0.65451 +
Diabetes_present*0.57367 - 23.9802
      Risk=100 * (1 - 0.88936^exp(Risk_Factors))
    }else{
      Risk_Factors = (log(Age) * 3.06117) + (log(Total_cholesterol) * 1.12370) -
(log(HDL_cholesterol) * 0.93263) +
        (log(Systolic_blood_pressure) * 1.93303) + Cigarette_smoker*0.65451 +
Diabetes_present*0.57367 - 23.9802
      Risk=100 * (1 - 0.88936^exp(Risk_Factors))
    }
  }else{
    if(is_sbp_under_treatment==1){
      Risk_Factors = (log(Age) * 2.32888) + (log(Total_cholesterol) * 1.20904) -
(log(HDL_cholesterol) * 0.70833) +
        (log(Systolic_blood_pressure) * 2.82263) + Cigarette_smoker*0.52873 +
Diabetes_present*0.69154 - 26.1931
      Risk=100 * (1 - 0.95012^exp(Risk_Factors))
    }else{
      Risk_Factors = (log(Age) * 2.32888) + (log(Total_cholesterol) * 1.20904) -
(log(HDL_cholesterol) * 0.70833) +
        (log(Systolic_blood_pressure) * 2.76157) + Cigarette_smoker*0.52873 +
Diabetes_present*0.69154 - 26.1931
      Risk=100 * (1 - 0.95012^exp(Risk_Factors))
    }
  }
  return(Risk)
}
```

```
# calculating the Framingham risk score using BMI
frisk_bmi_calc <- function(gender, Age, bmi,
                          Systolic_blood_pressure, is_sbp_under_treatment, Cigarette_smoker,
Diabetes_present){
  if(gender=="M" || gender=="m"){
    if(is_sbp_under_treatment==1){
      Risk_Factors = (log(Age) * 3.11296) + (log(bmi) * 0.79277) +
        (log(Systolic_blood_pressure) * 1.92672) + Cigarette_smoker*0.70953 +
Diabetes_present*0.53160 - 23.9388
      Risk=100 * (1 - 0.88431^exp(Risk_Factors))
    }else{
      Risk_Factors = (log(Age) * 3.11296) + (log(bmi) * 0.79277) +
        (log(Systolic_blood_pressure) * 1.85508) + Cigarette_smoker*0.70953 +
Diabetes_present*0.53160 - 23.9388
      Risk=100 * (1 - 0.88431^exp(Risk_Factors))
    }
  }else{
    if(is_sbp_under_treatment==1){
      Risk_Factors = (log(Age) * 2.72107) + (log(bmi) * 0.51125) +
        (log(Systolic_blood_pressure) * 2.88267) + Cigarette_smoker*0.61868 +
Diabetes_present*0.77763 - 26.0145
      Risk=100 * (1 - 0.94833^exp(Risk_Factors))
    }else{
      Risk_Factors = (log(Age) * 2.72107) + (log(bmi) * 0.51125) +
        (log(Systolic_blood_pressure) * 2.81291) + Cigarette_smoker*0.61868 +
Diabetes_present*0.77763 - 26.0145
      Risk=100 * (1 - 0.94833^exp(Risk_Factors))
    }
  }
  return(Risk)
}
```
